# An Exploration of Mutagenesis in a Family with Cleidocranial Dysplasia without *RUNX2* Mutation

**DOI:** 10.3389/fgene.2021.748111

**Published:** 2021-10-19

**Authors:** Dandan Liu, Yang Liu, XianLi Zhang, Yixiang Wang, Chenying Zhang, Shuguo Zheng

**Affiliations:** ^1^ National Engineering Laboratory for Digital and Material Technology of Stomatology, Department of Preventive Dentistry, National Center of Stomatology, National Clinical Research Center for Oral Diseases, Peking University School and Hospital of Stomatology, Beijing, China; ^2^ Department of Stomatology, Xuanwu Hospital Capital Medical University, Beijing, China; ^3^ National Engineering Laboratory for Digital and Material Technology of Stomatology, Central Laboratory, Department of Oral and Maxillofacial Surgery, National Center of Stomatology, National Clinical Research Center for Oral Diseases, Peking University School and Hospital of Stomatology, Beijing, China

**Keywords:** cleidocranial dysplasia, RUNX2, IGSF10, osteoblast differentiation, mutation analysis

## Abstract

Cleidocranial dysplasia (CCD) is an autosomal dominant inheritable skeletal disorder characterized by cranial dysplasia, clavicle hypoplasia, and dental abnormalities. Mutations involving Runt-related transcription factor 2 (*RUNX2*) are currently the only known molecular etiology for CCD but are not identified in all CCD patients. No *RUNX2* abnormality can be detected in about 20–30% of patients, and the molecular cause remains unknown. The present study includes a family case with typical features of CCD. *RUNX2* mutation was first screened by sequencing analysis, and no mutation was detected. Copy number alterations of the *RUNX2* gene were then measured by quantitative PCR and multiplex ligation-dependent probe amplification (MLPA). No copy number variation in *RUNX2* could be detected. We performed whole-exome sequencing (WES) to identify the underlying genetic mutations. Unexpectedly, no abnormalities could be detected in genes related to the RUNX2 signaling pathway. Therefore, it was supposed that other new unknown gene variations might contribute to the CCD phenotype. We focused on Immunoglobulin superfamily member 10 (*IGSF10*), a gene related to bone development. An *IGSF10* frameshift mutation (c.6001_6002delCT, p.Leu2001Valfs*24) was detected by WES. Sanger sequencing verified that this mutation was only detected in the patient and her affected mother but not in her unaffected father. Bioinformatics studies demonstrated that this mutation could change the 3D structure of the IGSF10 protein and severely damage its function. In addition, alkaline phosphatase (ALP) activity and the ability to form mineralized nodules were inhibited by IGSF10 knockdown compared with normal controls. The expression of bone sialoprotein (BSP) was significantly reduced by IGSF10 knockdown, but not that of other osteogenic markers. Our results provide new genetic evidence that *IGSF10* mutation might contribute to CCD.

## Introduction

Cleidocranial dysplasia (CCD; MIM 119600) is an autosomal dominant skeletal disorder that is, characterized by delayed closure of the fontanels, hypoplastic or aplastic clavicles, and dental anomalies (Mundlos, 1999). Mid-face hypoplasia, hand abnormalities, short stature, and other skeletal anomalies are also common (Mundlos, 1999). Highly variable expressivity generates phenotypic heterogeneity among CCD patients, even within the same family (Chitayat et al., 1992), which adds to the difficulty of diagnosing CCD.

The causative gene of CCD has been identified as Runt-related transcription factor 2 (*RUNX2*, also known as *CBFA1*) (Mundlos et al., 1997), which is essential for osteoblast differentiation and skeletal development (Komori, 2020). Numerous mutations in *RUNX2* have been identified in patients with CCD, the majority of which were missense, nonsense, and frameshift mutations (Zhou et al., 1999; Otto et al., 2002). Chromosomal abnormalities, splicing mutations, and intragenic deletions/duplications were also identified in some patients (Lee et al., 2008; Purandare et al., 2008; Ott et al., 2010; Northup et al., 2011; Zhang et al., 2019). However, *RUNX2* mutations are only detected in approximately two-thirds of patients with a CCD phenotype (Mundlos et al., 1997; Baumert et al., 2005), and numerous CCD patients have been identified who had no detectable mutations in *RUNX2* by sequencing. There are several possible reasons for this observation. First, genetic heterogeneity may exist, such as mutation in *RUNX2* gene regulatory elements or in genes encoding proteins that interact with RUNX2, including core-binding factor subunit beta (*CBFB*) (Goto et al., 2004; Napierala et al., 2005; Khan et al., 2006). Furthermore, other genetic diseases can show a similar phenotype to that of CCD, such as parietal foramina with cleidocranial dysplasia, which results from mutations in Drosophila muscle segment homeobox gene homologue 2 (*MSX2*) (Garcia-Minaur et al., 2003; Ott et al., 2012).

Among patients with normal *RUNX2* sequencing analysis results, detection of *RUNX2* copy number changes identified a microdeletion/duplication frequency of 26% (Ott et al., 2010). However, for the remaining patients the molecular cause is unknown. This indicates that other unreported mechanisms may contribute to CCD.

In this study, we present a familial case with typical features of CCD. However, sequencing analysis did not reveal any mutations in *RUNX2*, and no microdeletion/duplication in *RUNX2* was detected by copy number analysis. Further exploration by whole-exome sequencing (WES) and bioinformatics analyses were performed and a new CCD candidate gene was identified, immunoglobulin superfamily member 10 (*IGSF10*), a member of the immunoglobulin superfamily. A role for IGSF10 in CCD has not been previously reported, though pathogenic *IGSF10* mutations have been detected in self-limited delayed puberty (DP) and congenital hypogonadotropic hypogonadism (CHH) (Howard et al., 2016; Amato et al., 2019). The primary manifestation of self-limited DP and CHH is delayed puberty, absent in the present patients (Amato et al., 2019; Raivio and Miettinen, 2019). After identification of the mutation, we preliminarily explored the role of IGSF10 in osteogenic differentiation.

## Materials and Methods

### Participants

Two CCD patients in a Chinese family were recruited according to the criteria for the clinical diagnosis of CCD (Mundlos, 1999), together with 50 control subjects. Informed consent was obtained from the guardians of the proband and other participants. All study protocols were approved by the Ethical Committee of Peking University Health Science Center (Approval Number: IRB00001052-07100).

### Mutation Analysis

Peripheral blood samples were collected from the participants and genomic DNA was extracted using a TIANamp Blood DNA mini kit (TIANGEN, Beijing, China) following the manufacturer’s instructions. *RUNX2* mutation was analyzed in the proband and her parents. The exons and exon-intron boundaries of *RUNX2* were amplified by polymerase chain reaction (PCR) using intron–exon specific primers as described previously (Quack et al., 1999). We also used other previously reported primers (Napierala et al., 2005) and designed others using software (Primer Premier 5 software, Premier Biosoft, Palo Alto, CA, United States) to amplify the 2.0 kb region upstream of the transcription start site of *RUNX2* transcript variant 1 (NM_001024630.4). To confirm *IGSF10* mutation detected in WES as pathogenic, we sequenced this variant in the proband, her parents and 50 unrelated control people. *IGSF10*-specific primers were also designed with the same software (Primer Premier 5 software, Premier Biosoft). Primer sequences are listed in Supplementary Table S1. Genomic DNA was amplified with Premix Taq™ (Takara, Shiga, and Japan). In brief, PCR reactions were performed with a DNA Engine PTC-200 (Bio-Rad Laboratories, Hercules, CA, United States) using a program described elsewhere (Zhang et al., 2017). The amplification products were bi-directionally sequenced using an ABI 3730 XL automatic sequencer (Applied Biosystems, Foster City, CA, United States). DNA sequences were analyzed using NCBI databases and the BLASTN (BLAST nucleotide) program (http://blast.ncbi.nlm.nih.gov/).

### Real-Time PCR

*RUNX2* copy number was determined by real-time PCR using a SYBR Green PCR kit (Roche Applied Science, Indianapolis, IN, United States). We detected the copy number of the coding region and the flanking untranslated regions (UTRs) of *RUNX2* using the primers and method described previously (Ott et al., 2010). The primers were listed in Supplementary Table S2. qPCR was performed in a total volume of 12 µl containing 10 ng genomic DNA, 6 µl FastStart Universal SYBR Green PCR Master (Roche Applied Science), and 1 µl primers (0.2 µmol each). Samples were run on the ABI 7,500 Real-Time PCR System (Applied Biosystems, Foster, CA, United States) in triplicate in separate reactions to determine the variation in copy number between CCD patients and controls. Two controls were analyzed. The copy number of *RUNX2* was normalized against those of the Albumin (*ALB*) gene and were calculated using the 2^−ΔΔCt^ method.

Real-time PCR was also used to evaluate the expression of osteogenic-associated genes. MC3T3-E1 cells are osteoblast precursor cells derived from mouse, we used this cell line to explore the role of IGSF10 in osteogenesis. After MC3T3-E1 cells were cultured in osteogenic medium (OM) for 7 days, total RNA was extracted using Trizol reagent (Invitrogen, Carlsbad, CA, United States) following the manufacturer’s instructions. Next, 500 ng of total RNA were used in a 10 µl reaction volume to synthesize cDNA using a PrimeScript RT reagent kit (Takara). The resultant cDNAs were amplified using specific sets of primers for *Igsf10*, *Runx2*, bone sialoprotein (*Bsp*), alkaline phosphatase (*Alp*), osteocalcin (*Ocn*), and osterix (*Osx*). The housekeeping gene glyceraldehyde-3-phosphate dehydrogenase (*Gapdh*) was used to normalize RNA expression levels. The primers used are listed in Supplementary Table S3. Primer specificity was confirmed with amplicon dissociation curves. The RT-qPCR was performed using 1 µl cDNA product in a 20 µl reaction volume with a SYBR Green PCR kit (Roche Applied Science) according to the manufacturer’s protocol, and the results were assessed with 7,500 Software 2.0.1 (Applied Biosystems). Relative mRNA expression was calculated using the 2^−ΔΔCt^ method with the formula:

$$F=2^{-\Delta \Delta \mathrm{Ct}}=\frac{2^{\left[\mathrm{Ct}\left(\mathrm{target gene}\right)-\mathrm{Ct}\left(\mathrm{GAPDH}\right)\right]\mathrm{control}}}{2^{\left[\mathrm{Ct}\left(\mathrm{target gene}\right)-\mathrm{Ct}\left(\mathrm{GAPDH}\right)\right]\mathrm{treatment}}}.$$

### MLPA and Karyotype Analysis

Multiplex ligation-dependent probe amplification (MLPA) was performed with the SALSA MLPA Probemix P080-C2 kit according to the manufacturer’s instructions (MRC-Holland, Amsterdam, Netherlands) by the Department of Medical Genetics, Peking University Health Science Center (Beijing, China). All the peak areas were normalized, and a ratio between 0.8 and 1.2 was considered normal. A heterozygous duplication was identified with a ratio between 1.3 and 1.7, and a heterozygous deletion between 0.3 and 0.7.

Karyotype analysis was performed by Giemsa-Trypsin (GTG) banding using standard procedures by the Department of Medical Genetics, Peking University Health Science Center (Beijing, China). Twenty G-banded metaphase cells were examined.

### WES and Data Analysis

Whole-exome sequencing (WES) was performed using genomic DNA from the proband and her parents. DNA libraries were prepared using the Agilent SureSelect system (Agilent Technologies, Santa Clara, CA, United States) and sequenced on the Illumina HiSeq 2000 platform (Illumina, San Diego, CA, United States) to generate 100 bp paired-end reads. Bioinformatics analyses included production of clean data, alignment to human reference genome hg19 using Burrows–Wheeler Aligner (BWA) (Li and Durbin, 2009), variant calling, and annotation using Samtools, Genome Analysis Tool Kit, and Annovar (Hintzsche et al., 2016). We then used in-house AnnoDB software to annotate the confident variant results. Quality control was present throughout the whole procedure. Next, steps were performed to identify potential causal variants. First, the variants were segregated by trait (variants present in affected individuals but not in unaffected family members). Second, variants were further filtered by minor allele frequency (MAF <0.5%) in the context of heterozygous genotype in the 1,000 Genomes database (1000G_ALL and 1000G_EAS) and the NHLBI exome variant server (ESP_ALL). Third, deleterious variants were picked based on SIFT, Polyphen-2, and variant impact. Then, the functions of those genes retained after filtering were reviewed, and genes related to bone development, especially those in the RUNX2 pathway, were selected for further study.

### Prediction of Damaging Effects

Multiple sequence alignment for IGSF10 was performed using ClustalW in MEGA-X software to analyze the conservation of affected amino acids (Kumar et al., 2018). To analyze the effect of the mutation on the molecular structure of IGSF10, the three-dimensional (3D) structures of wild-type and mutant IGSF10 were predicted *in silico* using I-TASSER (https://zhanglab.ccmb.med.umich.edu/I-TASSER/) (Yang et al., 2015).

### Cell Culture

MC3T3-E1, a pre-osteoblast murine cell line, was purchased from the Cell Bank of Chinese Academy of Sciences and cultured in a proliferation medium containing α-MEM (Gibco, Grand Island, NY, United States), 10% fetal bovine serum (Gibco), 100 U/ml penicillin (Gibco) and 100 μg/ml streptomycin (Gibco) in an incubator at 37°C with 5% CO_2_. For osteogenic induction, MC3T3-E1 cells were cultured in proliferation medium plus 100 nM dexamethasone (Sigma-Aldrich, St Louis, MO, United States), 10 mM β-glycerophosphate (Sigma-Aldrich), and 50 μg/ml l-ascorbic acid (Sigma-Aldrich).

### Transfection

The pCMV5-*Runx2* plasmid expressing full-length wild-type RUNX2 was provided by the School of Dentistry, University of Michigan (Ann Arbor, MI, United States). Plasmid pcDNA3-CMF608FL (Segev et al., 2004), containing a full-length open reading frame for expression of Flag-tagged rat IGSF10 protein, was provided by Quark Pharmaceuticals Inc. (Newark, CA, United States/Ness Ziona, Israel). The sequences of wild-type *Runx2* and *Igsf10* were confirmed by DNA sequencing. Empty pCMV5 and pcDNA3.1 vectors served as transfection controls. Three small interfering RNAs (siRNAs) for *Igsf10* and *Runx2* were purchased from Ribo Bio Technology (Guangzhou, China). The most efficient siRNA for each gene was selected for the subsequent experiments: si-Igsf10, 5′-GCA​CCT​TCC​TGA​TTT​CAA​A-3′; and si-Runx2, 5′-GCA​CGC​TAT​TAA​ATC​CAA​A-3′. Scrambled siRNA was used as a negative control.

For transfection, MC3T3-E1 cells were seeded at a density of 1×10^5^ cells/well in six-well plates. When cells reached 80% confluency for plasmids and 30–50% confluency for siRNAs, transfection was performed with Lipofectamine 3,000 (Invitrogen, Shanghai, China) in accordance with the manufacturer’s instructions. Cells were collected for subsequent experiments 48 h after transfection.

### Construction and Transfection of Lentiviral Igsf10-shRNA Expression Vector

For IGSF10 knockdown, we used a lentiviral RNAi small hairpin RNA (shRNA)-encoding system using the pHBLV-U6-Puro lentiviral RNAi vector (Hanbio, Shanghai, China). The targeting sequence of the *Igsf10* shRNA was 5′-GCA​CCT​TCC​TGA​TTT​CAA​A-3′. To establish stable IGSF10-knockdown cell lines, MC3T3-E1 cells were transduced with the lentiviral RNAi vector at a multiplicity of infection (MOI) of approximately 100 in the presence of 10 μg/ml polybrene (Hanbio), and infected cells were selected with 2 μg/ml puromycin (Sigma-Aldrich). The empty lentivector was used as a negative control.

### ALP Staining and ALP Activity Assay

MC3T3-E1 cells were cultured in OM 12-well plates for 7 days. Then, the cells were fixed with 4% paraformaldehyde for 15 min. After washing in double-distilled water (ddH_2_O) three times, the cells were stained using an ALP histochemical staining kit (Cwbiotech, Beijing, China) in accordance with the manufacturer’s protocol.

For ALP activity testing, MC3T3-E1 cells were collected after osteogenic induction for 7 days. The cells were lysed in RIPA buffer containing protease and phosphatase inhibitors (Huaxing bio, Beijing, China). Lysates were centrifuged at 13,523 × *g* at 4°C for 30 min and the supernatants were collected for analysis. Protein concentration was determined using a Pierce BCA protein assay kit (Thermo Fisher Scientific, Waltham, MA, United States). ALP activity was measured with an ALP activity kit (Nanjing Jiancheng Biotech, Nanjing, China) in accordance with the manufacturer’s instructions.

### Alizarin Red Staining and Quantification

MC3T3-E1cells were fixed in 4% paraformaldehyde after osteogenic induction. Alizarin red staining was performed using 2% alizarin red (Sigma-Aldrich). For quantitative assessment of the degree of mineralization, the stain was dissolved in 10% cetylpyridinium chloride (Sigma-Aldrich) and the absorbance at 562 nm was measured.

### Western Blotting

After MC3T3-E1 cells were cultured in OM for 7 days, total protein was extracted and quantified with the method described in the ALP activity assay. Samples with equal amounts of denatured protein were analyzed by sodium dodecyl sulphate polyacrylamide gel electrophoresis. Proteins were transferred to polyvinylidene difluoride membranes (Millipore, Bedford, MA, United States) and blocked in 5% skimmed milk. Then, membranes were incubated with primary antibodies against RUNX2 (CST, Danvers, MA, United States), BSP (CST), OSX (Abcam, Cambridge, United Kingdom), and GAPDH (Huaxing bio) at 4°C overnight. After washing in TBS-T, the membranes were incubated with HRP-conjugated secondary antibody (Huaxing bio) for 1 h at room temperature and visualized using an enhanced chemiluminescence blotting kit (Cwbiotech).

### Statistical Analysis

All experiments were repeated at least three times and data are expressed as the mean ± SD. Differences between groups were determined by statistical analysis with GraphPad Prism v8.02 (GraphPad Software, Bethesda, MD, United States). Normal distribution and variance homogeneity tests were carried out. For the expression of IGSF10 in the osteogenesis of MC3T3-E1 cells, one-way analysis of variance (ANOVA) was performed and followed by Bonferroni’s post hoc test. Comparison between two groups was performed using unpaired Student’s t-test. *p* < 0.05 was considered statistically significant.

## Results

### Clinical Features of the Cleidocranial Dysplasia Patients

**FIGURE 1:**
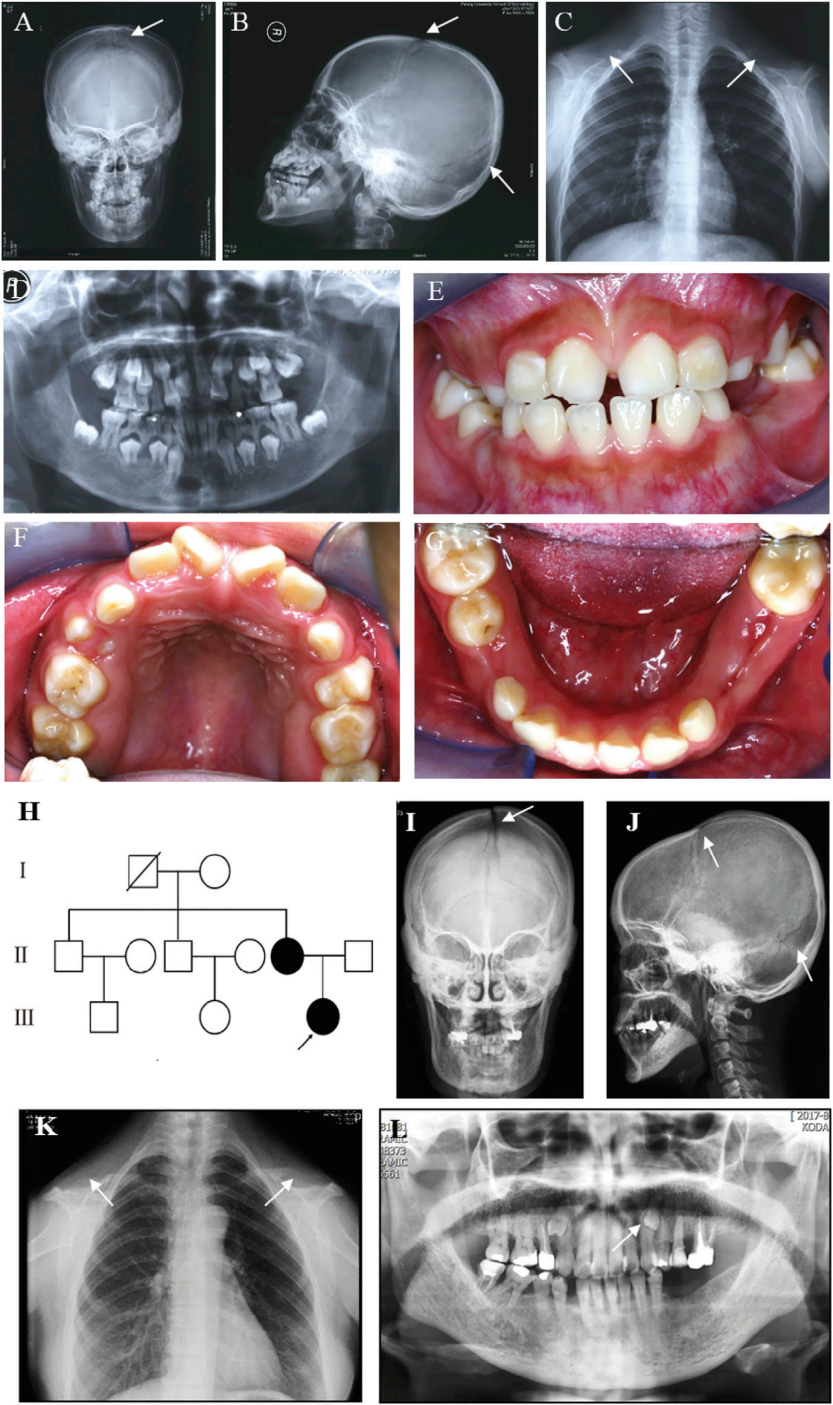
Clinical and radiographic findings of the proband and her affected mother. **(A)** Anteroposterior and **(B)** Lateral radiographs of the skull of the proband show patent frontal fontanelle and multiple Wormian bones. **(C)** Chest radiograph of the proband shows cone-shaped thorax and bilateral aplasia of clavicles. **(D–G)** Panoramic radiograph and Intraoral photographs of the proband show dental anomalies, including retained deciduous teeth and delayed eruption of permanent teeth. **(H)** Pedigree of the family. The arrow indicates the proband. **(I)** Anteroposterior and **(J)** Lateral radiographs of the skull of the proband’s mother show patent frontal fontanelle and multiple Wormian bones. **(K)** Chest radiograph of the proband’s mother shows cone-shaped thorax and bilateral aplasia of clavicles. **(L)** Panoramic radiograph of the proband’s mother shows dental anomalies, such as supernumerary teeth in the apices between the left upper canine and the first premolar. The typical signs of patients are pointed out by white arrows.

A Chinese family was studied. The proband, a 12-year-old girl, showed typical features of CCD, including patent fontanelles and bilateral hypoplastic clavicles on skull and chest radiography (Figures 1A–C). Furthermore, multiple dental anomalies commonly seen in CCD were also found, such as retained deciduous teeth, delayed eruption of permanent teeth, and crossbite (Figures 1D–G). A narrowing upper dental arch and high vault were also present (Figure 1F). Notably, supernumerary teeth were absent.

The pedigree of the family showed that the proband’s mother was also a CCD patient (Figure 1H). The phenotype of the proband’s mother was similar to the proband (Figure 1I–L), except that supernumerary teeth were present in the proband’s mother (Figure 1L). However, the number of supernumerary teeth was uncertain because the tooth extraction history of the mother was not reliable. No obvious signs of any other health problems were present in the two patients. After a detailed inquiry of the family medical history, we found that the proband’s two uncles and grandparent had no obvious CCD phenotypes. We were unable to confirm the genotype because they refused to carry out genetic testing.

### No *RUNX2* Mutations Were Detected

**FIGURE 2:**
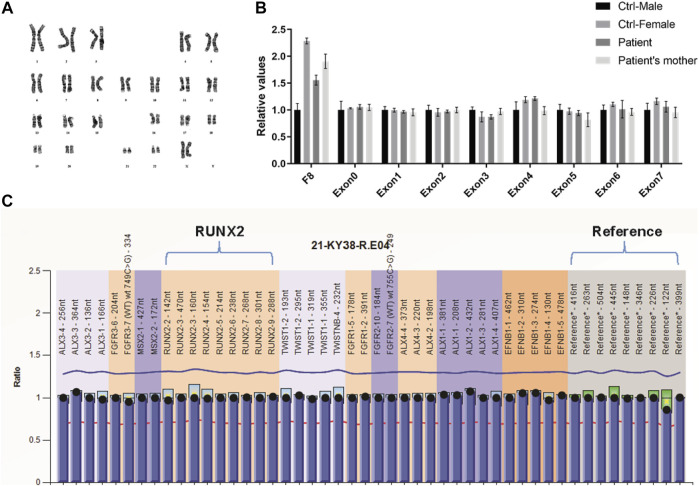
Variation analysis of *RUNX2* gene for the family CCD patients. **(A)** GTG-banding karyotype of lymphocytes of the proband reveals a normal karyotype of 46 chromosomes. **(B, C)** Analysis of *RUNX2* copy number using RT-qPCR **(B)** and MLPA **(C)** shows a normal *RUNX2* copy number.

Sequencing analysis was first performed for the coding region, exon–intron boundaries, and the 2.0 kb region upstream of the transcription start site of *RUNX2* by PCR and direct sequencing to explore the genetic basis of the familial case. However, no abnormality was found (data not shown). Because copy number variation of *RUNX2* can cause CCD (Lee et al., 2008), we screened for intragenic deletions and duplications of *RUNX2* by real-time PCR and MLPA. However, we detected no causative copy number variant of *RUNX2* (Figures 2B,C). Chromosomal abnormalities involving *RUNX2* can also result in CCD (Purandare et al., 2008), and therefore karyotype analysis was performed for the proband. A normal karyotype was shown for the proband (Figure 2A); therefore, we speculated that genetic variations in genes other than *RUNX2* may lead to the disease.

### Detection of an *IGSF10* Mutation by WES

**FIGURE 3:**
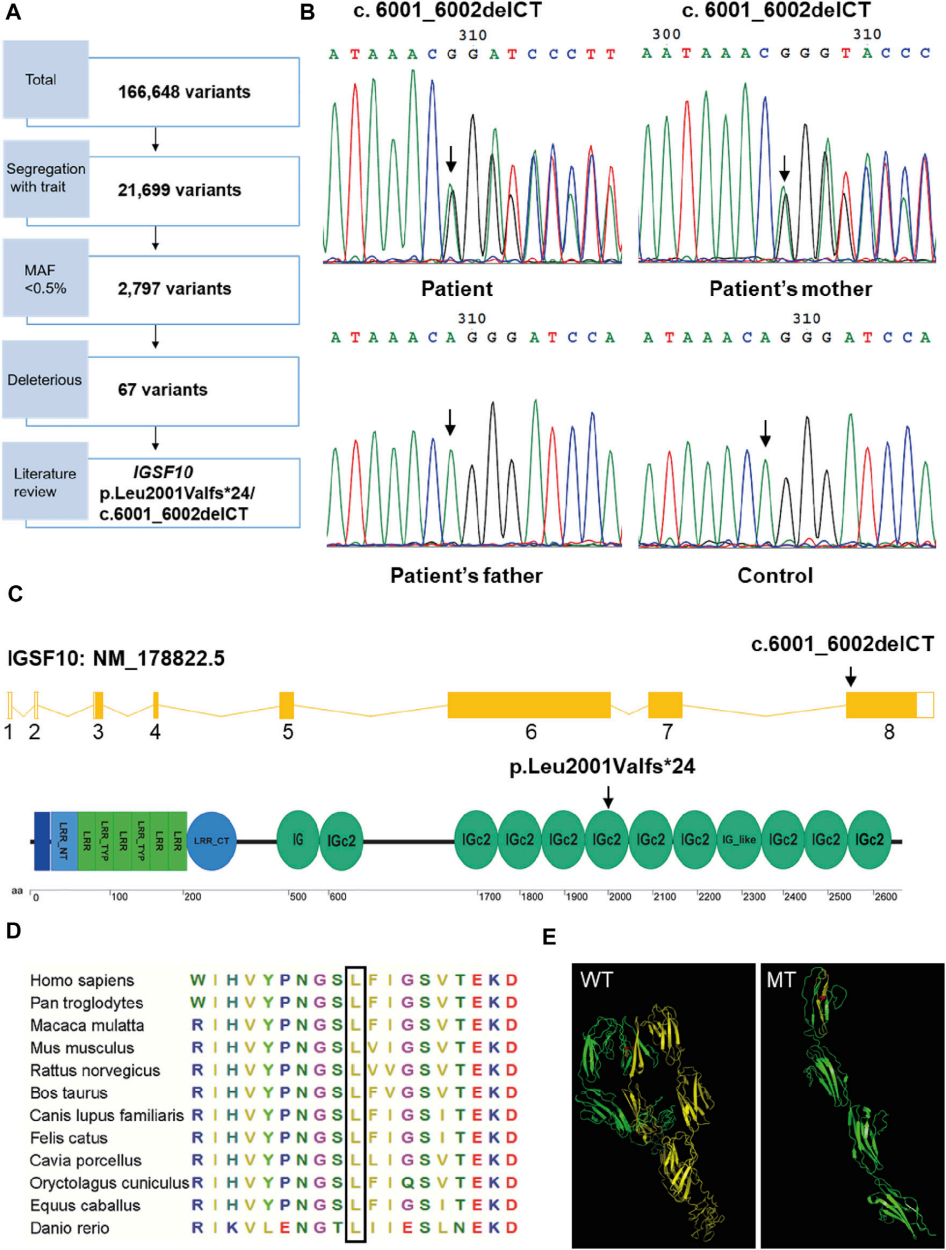
Mutation analysis of *IGSF10* gene for the CCD patients. **(A)** Flowchart of whole-exome sequencing filtering outcomes. First, only variants that were found in all affected subjects in the family were retained. From among these segregating variants, only those with minor allele frequency (MAF) less than 0.005 in the 1,000 Genomes database and the NHLBI exome variant server were selected for further analysis. Deleterious variants were then picked out based on the SIFT, Polyphen-2 and variant impact. *IGSF10* was scanned out by further literature review. **(B)** Identification of the heterozygous mutation in *IGSF10* gene in the family of the proband and unaffected controls. The mutated sequence (upper panel) and control sequence (lower panel) were shown, respectively. Arrows indicated the mutation site. **(C)** Structure of the IGSF10 transcript and protein. Arrows indicated the mutation site. **(D)** Partial amino acid sequences alignment in IGSF10 protein among twelve species. The positions of the mutated amino acid in our study are indicated using a black box. **(E)** The three-dimensional structure of IGSF10 predicted by I-TASSER in wild-type and mutated IGSF10. WT shows the normal last 10 Ig domains, and MT displays the mutated form corresponding to WT. Yellow means the structure from the 2001th aa to the final aa. Red represents the 2001th aa.

To identify the genetic cause of the familial case, we performed WES and bioinformatics analyses for the proband and her parents. WES identified 166,648 variants (Figure 3A) and a total of 67 candidate genes were identified after filtering for a MAF <0.5%, predicting deleterious variants, and segregating with the trait in affected family members (Supplementary Table S4). These 67 candidate genes were assessed by a literature review. Unexpectedly, no variants were detected in genes related to RUNX2 signaling. Therefore, we predicted that variation in an unknown gene may contribute to CCD in this family. We found that, of the 67 candidate genes, only *IGSF10* was related to osteogenesis based on current knowledge. *IGSF10* was finally selected for further investigation because of its potential role in bone development (Segev et al., 2004).

A heterozygous two-base deletion in *IGSF10* (c.6001_6002delCT, p.Leu2001Valfs*24) was detected in the proband and her mother (Supplementary Table S4). 50 unaffected controls were analyzed by PCR and Sanger sequencing to verify the mutation as pathogenic. Consistent with the WES data, a heterozygous two-base deletion of *IGSF10* was detected only in the proband and her affected mother, but not in normal controls or her unaffected father (Figure 3B). This novel mutation was in exon 8 of *IGSF10* (NM_178,,822.5) and resulted in a frameshift from codon 2001 to the resultant premature stop codon 2024. This was predicted to encode an IGSF10 protein truncated at the sixth immunoglobulin-like domain (Ig domain) with the loss of 600 amino acids from the C-terminus (Figure 3C).

Partial amino acid sequence alignment of the IGSF10 protein from 12 species showed that the affected amino acid, L2001, has a high level of evolutionary conservation among species (Figure 3D). Furthermore, the detected mutation was predicted by I-TASSER to alter the three-dimensional structure of the last ten Ig domains of IGSF10 (Figure 3E), leading to a loss of function of the predicted protein.

### IGSF10 Induces Osteogenic Differentiation and Regulates Bone Sialoprotein

**FIGURE 4:**
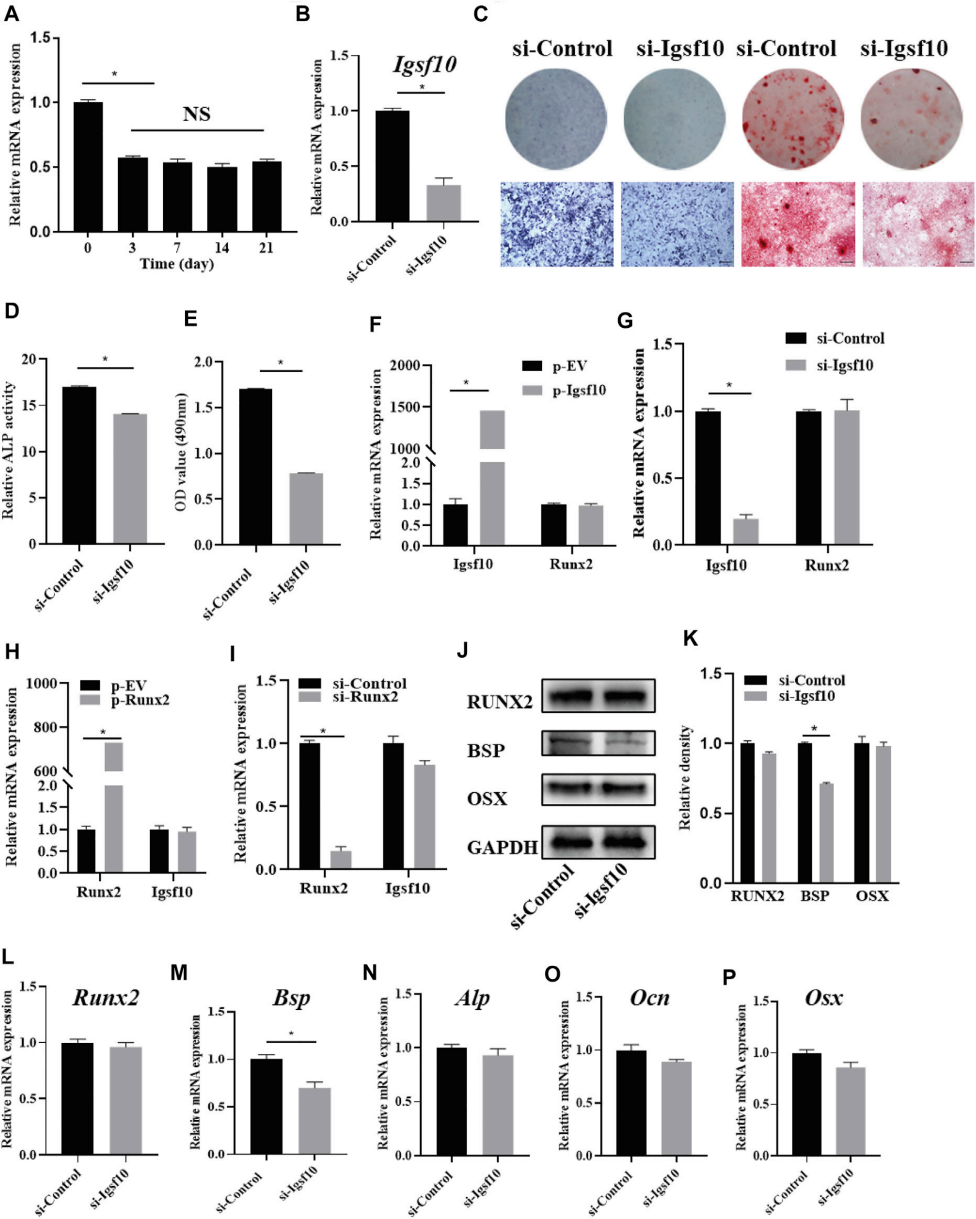
The effect of IGSF10 on the osteogenic capacity of MC3T3-E1 cells. **(A)** The expression of *Igsf10* in the osteogenesis of MC3T3-E1 cells was detected by real-time PCR. **(B)** The efficiency of *Igsf10* knockdown in MC3T3-E1 cells was confirmed by real-time PCR. **(C)** ALP staining and Alizarin red staining after induction in osteogenic medium (OM). The bottom row indicates microscopic views. Scale bar, 500 μm. **(D)** ALP activity was analyzed after induction in OM. **(E)** Quantification of alizarin red staining by spectrophotometry. **(F,G)** The level of *Runx2* expression after *Igsf10* overexpression **(F)** or knockdown **(G)** transiently. **(H,I)** The expression of *Igsf10* after *Runx2* overexpression **(H)** or knockdown **(I)**. **(J,K)** Western blotting and quantification of RUNX2, BSP, and OSX in MC3T3-E1 cells with stable *Igsf10* knockdown after cultured in OM. **(L–P)** Quantitative analysis of the mRNA levels of *Runx2*, *Bsp*, *Alp*, *Ocn*, and *Osx* in MC3T3-E1 cells with stable *Igsf10* knockdown after cultured in OM. NS: not significant. *p < 0.05.

The role of IGSF10 in osteogenic differentiation was further explored. We evaluated the expression dynamics of *Igsf10* mRNA after osteogenic induction in MC3T3-E1 cells and found that the levels of *Igsf10* mRNA decreased upon OM treatment (Figure 4A). We then constructed an MC3T3-E1 cell line with stable IGSF10 knockdown. IGSF10 knockdown efficiency was first assessed by real-time PCR. The mRNA level of *Igsf10* was decreased by more than 60% in MC3T3-E1 cells compared with control cells (Figure 4B). Regrettably, the level of IGSF10 protein could not be determined because a reliable IGSF10 antibody was not available.

We incubated stable IGSF10-knockdown cells and control cells (named si-Igsf10 and si-Control, respectively) in OM medium and analyzed their osteogenic ability. ALP activity (Figures 4C,D) and the ability to form mineralized nodules (Figures 4C,E) were obviously impaired in IGSF10-knockdown cells compared with control cells.

RUNX2 is a key protein in osteogenesis; therefore, we asked whether the role of IGSF10 in osteogenesis is mediated by RUNX2. Unexpectedly, the level of *Runx2* expression did not change, irrespective of overexpression (Figure 4F) or knockdown of IGSF10 (Figure 4G). Similarly, *Igsf10* expression was not affected by RUNX2 overexpression (Figure 4H) or knockdown (Figure 4I).

The effect of IGSF10 knockdown on other osteogenic-associated genes was subsequently explored. The level of *Bsp* mRNA decreased by 30% after IGSF10 knockdown (Figure 4M). The levels of RUNX2 and BSP proteins were similar to their mRNA levels after knockdown of IGSF10, with the level of BSP protein showing a slight reduction (Figures 4J,K). The mRNA levels of *Alp*, *Ocn*, and *Osx* showed no significant change after IGSF10 knockdown in MC3T3-E1 cells (Figures 4N–P).

## Discussion

We studied the genetic basis of typical CCD in a family pedigree. We did not detect any sequence or copy number variation in *RUNX2* by direct sequencing and copy-number analyses. However, we identified a new candidate gene for CCD, *IGSF10*, and explored its role in osteogenesis.

The diagnosis of CCD mainly depends on clinical observation and imaging examination. Detection of a pathogenic gene plays an auxiliary role when the clinical characteristics are not typical. The main clinical features of CCD include persistently open skull sutures with bulging calvaria, hypoplasia or aplasia of the clavicles permitting abnormal facility in apposing the shoulders, wide pubic symphysis, short middle phalanx of the fifth fingers, and multiple dental anomalies, such as retained deciduous teeth, delayed eruption of permanent teeth, and supernumerary teeth (Mundlos, 1999). As an autosomal dominant inherited disease with complete penetrance, the generally accepted causative gene of CCD is *RUNX2*. However, the expressivity of CCD is variable, even among different family members (Chitayat et al., 1992). Three different subtypes are defined for CCD, including classic, mild, and isolated dental anomalies (Zhou et al., 1999). Classic CCD patients show hypoplastic clavicles and delayed closure of the anterior fontanelle, in addition to characteristic craniofacial features. The isolated dental CCD phenotype only shows dental anomalies without cranial or axial skeletal anomalies. The phenotype of mild CCD is between classic CCD and dental CCD, with nominal or absent clavicle findings.

CCD should be differentiated from other similar skeletal diseases. Pycnodysostosis and mandibuloacral dysplasia are two disorders that should be considered as a differential diagnosis of cleidocranial dysplasia. Pycnodysostosis (MIM 265800) also shows hypoplastic clavicles, delayed closure of fontanelles, and delayed tooth eruption. Features that differentiate pycnodysostosis from CCD are acro-osteolysis, bone sclerosis with tendency to fracture, absence of supernumerary teeth, and cathepsin K (*CTSK*) gene mutations (Elmore et al., 1966; Bizaoui et al., 2019). Mandibuloacral dysplasia (MIM 248370) is an autosomal recessive disorder characterized by growth retardation, craniofacial anomalies with mandibular hypoplasia, skeletal abnormalities with progressive osteolysis of the distal phalanges and clavicles, and pigmentary skin changes (Cenni et al., 2018). Typical progressive osteolysis of the distal phalanges and pigmentary skin changes make it easy to differentiate from CCD (Garavelli et al., 2009). Furthermore, Yunis–Varon syndrome (MIM 216340), Crane–Heise syndrome (MIM 218090), CDAGS syndrome (MIM 603116), and hypophosphatasia (MIM 241500) also need to be differentiated from CCD (Crane and Heise, 1981; Basel-Vanagaite et al., 2008) (Méhes et al., 1972; Zand et al., 2003; Mendoza-Londono et al., 2005). In the present study, the proband showed typical features of CCD, including patent fontanelles, bilateral hypoplastic clavicles, and multiple dental anomalies. Therefore, this patient was confidently diagnosed with CCD.

Heterozygous mutations in *RUNX2* have been identified as the cause of CCD (Mundlos et al., 1997). Heterozygous *Runx2* mutant mice also display all the representative features of CCD, including open fontanelles and hypoplastic clavicles, but not the dental anomalies because mice only have one set of teeth with no replacement dentition (Selby et al., 1993; Komori et al., 1997; Otto et al., 1997). To date, more than 233 heterozygous mutations in *RUNX2* have been identified in CCD patients, the majority of which are missense, nonsense, and frameshift mutations (Jaruga et al., 2016). Additionally, when sequence variants are not detected by direct sequencing, real-time PCR assays or MLPA are good tools to detect *RUNX2* gene deletion or duplication by analyzing copy number variants (Lee et al., 2008; Franceschi et al., 2015; Sun et al., 2016). However, in the present proband, no abnormality was detected by direct sequencing in the coding region, flanking intron sequences, or regulatory region of *RUNX2*. Furthermore, no *RUNX2* microdeletions/duplications were detected by real-time PCR or MLPA. Therefore, genetic heterogeneity or other genes in the RUNX2 pathway were alternative causative candidates for this familial case.

WES and bioinformatics analyses were used to explore the genetic basis of CCD in the present patient. We first focused on variants in genes involved in the RUNX2 pathway; however, our screen detected no pathogenic mutations. Subsequently, other potential candidate genes were analyzed and *IGSF10* was finally identified as the most promising candidate. IGSF10 (also known as CMF608) belongs to the immunoglobulin superfamily that contain one or more Ig domains, and can bind with other proteins (Natarajan et al.). IGSF10 is a mechanical strain-induced bone-specific protein that is, involved in maintaining the osteochondroprogenitor cell pool (Segev et al., 2004). We detected a heterozygous two-base deletion in exon 8 of *IGSF10* in the proband and her affected mother, resulting in a frameshift from codon 2001 to the resultant premature stop codon 2024. This leads to a truncated IGSF10 protein that has a severely affected three-dimensional structure. This is the first report of a mutation in *IGSF10* being associated with CCD, although deleterious variants of *IGSF10* have been described in DP and CHH patients (Howard et al., 2016; Amato et al., 2019). The primary manifestation of self-limited DP and CHH is delayed puberty, and no signs of delayed puberty or hypogonadism was shown in the proband following our subsequent follow-up. Interestingly, height and bone mineral density can be compromised in some adults with a history of DP (Zhu and Chan, 2017). Parker et al. also found that women with delayed puberty had a higher risk of osteoporosis (Parker et al., 2014). We therefore suggest that IGSF10 is involved in the pathogenesis of some bone-related diseases. However, the underlying mechanism is still unclear and needs further exploration.

IGSF10 plays an important role in bone development. Our research showed that *Igsf10* mRNA expression was down-regulated in OM-treated MC3T3-E1 cells, and the osteogenic ability of MC3T3-E1 cells was decreased by IGSF10 knockdown. Moreover, previous research showed that IGSF10 expression was significantly elevated *in vivo* in response to bone-formation-promoting stimuli such as estrogen administration, and downregulated in response to sciatic neurotomy. The expression of IGSF10 was also confined to skeletal progenitor cells abundant in the regions of active bone modeling and remodeling (Segev et al., 2004). Taking all these results together, we presume that high IGSF10 expression may be associated with a specific developmental stage of MC3T3-E1 cells, and IGSF10 expression may be a precondition to osteogenic differentiation rather than a downstream target.

As a known causative gene of CCD, the expression of *Runx2* after IGSF10 knockdown or overexpression was examined. Unexpectedly, IGSF10 over- or under-expression had no effect on the expression of *Runx2*, and vice versa. Previous studies showed different results. Overexpression of IGSF10 in ROS17/2.8 cells increased the expression of *Runx2* (Segev et al., 2004), while overexpression of RUNX2 in NIH3T3 cells downregulated *Igsf10* expression (Stephens and Morrison, 2014). There are two possible reasons for this phenomenon. First, the same treatment could result in different or contrasting results from different research backgrounds. In addition, other unknown regulatory mechanisms could be involved in IGSF10-mediated bone development.

In our further study, the expression of other osteoblast-related genes, including *Alp*, *Osx*, and *Ocn*, was not affected by IGSF10 knockdown, although the expression of *Bsp* was significantly decreased. BSP is a member of the “Small Integrin-Binding Ligand N-linked Glycoproteins” (SIBLING) extracellular matrix protein family of mineralized tissues (Bouleftour et al., 2016). It has long served as an early marker of osteoblast differentiation, as its expression is associated with the onset of mineralization (Chen et al., 1992; Midura et al., 2004). Taken together, these results indicate that the mechanisms by which IGSF10 regulates osteogenesis may be complex. One of the mechanisms may be that IGSF10 regulates early mineralization through pathways involving BSP.

In summary, the present study is a preliminary exploration of the genetic heterogeneity of CCD, and further studies need to be performed in the future to explore the underlying pathogenic mechanisms of *IGSF10* mutation. More CCD patients without *RUNX2* mutation need to be collected and their *IGSF10* mutation status determined. Also, animal *Igsf10*-knockout models need to be constructed to explore the role of IGSF10 in bone development and osteogenesis. Recently, variations in deep intronic sequence have been reported as a cause of monogenic disorders as well as hereditary cancer syndromes (Vaz-Drago et al., 2017). Deep intronic mutations (i.e., more than 100 base pairs away from exon-intron boundaries) could lead to pseudo-exon inclusion, disrupt transcription regulatory motifs and inactivate non-coding RNA genes (Dhir and Buratti, 2010; Romano et al., 2013; Szafranski et al., 2013; Jafarifar et al., 2014). However, so far, there is no report in CCD caused by the deep intron mutation of *RUNX2*. Due to the limitations of our current study, we will next detect the *RUNX2* deep intron in this family to further eliminate this possibility.

In conclusion, we report a familial case of CCD for whom no variation in *RUNX2* was detected by direct sequencing or copy-number analyses. A new candidate gene for CCD, *IGSF10*, was identified by WES. We explored the function of IGSF10 in osteogenesis and speculate that IGSF10 may regulate early osteogenic differentiation by targeting BSP. Our results provide new genetic evidence that *IGSF10* variation may contribute to CCD. While validating the link between IGSF10 and CCD, the mechanism by which *IGSF10* mutation leads to CCD needs further exploration.

## Data Availability

The original contributions presented in the study are publicly available. This data can be found here: https://www.ncbi.nlm.nih.gov/sra/PRJNA750609
