# Supplementary material for: An Exploration of Mutagenesis in a Family with Cleidocranial Dysplasia without RUNX2 Mutation
Source: Front Genet. 2021 Oct 19;12:748111. doi: 10.3389/fgene.2021.748111 (PMC8560734; doi:10.3389/fgene.2021.748111)
Supplement: Supplementary file 1 [file DataSheet1.docx]

**Tables:**

**Supplementary Table 1.** Primer sequences of mutation analysis in the study.

| **Gene name and region** | **Forward**  **primer** | **Reverse**  **primer** |
| --- | --- | --- |
| *RUNX2* Promoter 1-1 | CATACTACAGCAGCTACCTAC | AGGAATAATGCCCACAGACAG |
| *RUNX2* Promoter 1-2 | CTCCATCGCTCCCAACTGATG | CAGAGCTCTGCAGTTAAGGGC |
| *RUNX2* Promoter 1-3 | GCCACCCAGCAAATATGAAG | CTGCTTGCAGCCTTAAACTG |
| *IGSF10*-MT | ACAGTTTCCCTCATTGCTAA | TAATCTCCTTCCTCCGCTAC |

**Supplementary Table 2.** Primer sequences of detecting *RUNX2* copy number.

| **Gene name and region** | **Forward**  **primer** | **Reverse**  **primer** |
| --- | --- | --- |
| *RUNX2*-Exon1& 5'-UTR | AAGTGCGGTGCAAACTTTCT | GGTAGTGACCTGCGGAGATT |
| *RUNX2*-Exon2 | TCCTCCTTGCCCCTCATTTC | TCTACGGGAATACGCATCACAA |
| *RUNX2*-Exon3 | CCTCGGAGAGGTACCAGATG | CCGGAGCTCAGCAGAATAAT |
| *RUNX2*-Exon4 | CCTTGACCATAACCGTCTTCA | TCCCGAGGTCCATCTACTGT |
| *RUNX2*-Exon5 | ATTTAGGGCGCATTCCTCAT | AAGGACTTGGTGCAGAGTTCA |
| *RUNX2*-Exon6 | CCTACCTGAGCCAGATGACG | ATCGGTGATGGCAGGAAG |
| *RUNX2*-Exon7 | AGAGCTCATCCCCCTCATTT | ACTGAGAGTGGAAGGCCAGA |
| *RUNX2*-Exon8& 3'-UTR | AGACGGTCTCACTGCCTCTC | GATGGTCCCTAATGGTGTGG |
| *ALB*-Exon12 | TGTTGCATGAGAAAACGCCA | GTCGCCTGTTCACCAAGGAT |
| *F8*-Exon3 | CTACCATCCAGGCTGAGGTTTATG | CACCAACAGCATGAAGACTGACA |

**Supplementary Table 3.** Primer sequences of selected genes used in real-time PCR.

| **Gene** | **Forward primer** | **Reverse primer** |
| --- | --- | --- |
| *Gapdh* | AGGTCGGTGTGAACGGATTTG | TGTAGACCATGTAGTTGAGGTCA |
| *Igsf10* | ATTTCGGTACCTGACCTCCATC | CTGTGCAGCATGAGTAACTCCAG |
| *Runx2* | GATGACACTGCCACCTCTGAC | GGGATGAAATGCTTGGGAAC |
| *Bsp* | CAGGGAGGCAGTGACTCTTC | AGTGTGGAAAGTGTGGAGTT |
| *Alp* | CCAACTCTTTTGTGCCAGAGA | GGCTACATTGGTGTTGAGCTTTT |
| *Ocn* | GAGGACCATCTTTCTGCTCACTCT | TTATTGCCCTCCTGCTTGGA |
| *Osx* | CGCTTTGTGCCTTTGAAAT | CCGTCAACGACGTTATGC |

**Supplementary Table 4.** Details of the 67 genomic variants identified in the family by exome sequencing after filtering.

| **Gene** | **Genomic variant (GRCh37)** | **dbSNP** | **Predicted change** | **Impact** | **SIFT** | **PolyPhen2** | **RefSeq** |
| --- | --- | --- | --- | --- | --- | --- | --- |
| *OR4C45* | 11:48373748G>C | rs73453188 | p.K84N | MODERATE | N/A | N/A | NM_001005513.1 |
| *PRB3* | 12:11420501C>T | rs12811811 | p.P228S | MODERATE | N/A | N/A | NM_006249.5 |
| *CC2D1B* | 1:52821486G>C | N/A | p.M701I | MODERATE | N/A | N/A | NM_032449.2 |
| *KRTAP10-4* | 21:45994582G>C | N/A | p.G316A | MODERATE | N/A | N/A | NM_198687.2 |
| *ORC2* | 2:201778717A>T | N/A | splice acceptor variant | HIGH | N/A | N/A | NM_006190.4 |
| *TRDN* | 6:123837327G>A | N/A | p.G170E | MODERATE | N/A | N/A | NM_006073.3 |
| *BCLAF1* | 6:136582401T>A | rs62431282 | splice donor variant | HIGH | N/A | N/A | NM_014739.2 |
| *SUPT20HL1* | X:24381727G>A | rs1207580 | p.A284T | MODERATE | N/A | N/A | NM_001136234.1 |
| *CD24* | Y:21154466A>T | rs10465460 | p.T44S | MODERATE | N/A | N/A | NM_001291738.1 |
| *SEC22B* | 1:145115815A>C | rs202212505 | p.T192P | MODERATE | N/A | N/A | NM_004892.5 |
| *PKD1L3* | 16:72001135G>A | rs144950332 | p.R789Q | MODERATE | N/A | N/A | NM_181536.1 |
| *PKD1L3* | 16:72007329A>G | N/A | p.Q616R | MODERATE | N/A | N/A | NM_181536.1 |
| *SSPO* | 7:149505606T>A | N/A | p.S2995R | MODERATE | N/A | N/A | NM_198455.2 |
| *ADAM18* | 8:39564424G>T | rs183149445 | splice donor variant | HIGH | N/A | N/A | NM_014237.2 |
| *ZNF660* | 3:44635903C>T | rs147042905 | p.T73M | MODERATE | N/A | 0.989(D) | NM_173658.2 |
| *SYTL4* | X:99931059G>A | rs139707843 | p.R661H | MODERATE | N/A | 0.999(D) | NM_001129896.2 |
| *PDHX* | 11:34979129C>T | N/A | p.R181W | MODERATE | 0(D) | 0.761(P) | NM_003477.2 |
| *KLHL33* | 14:20898339C>T | N/A | p.R166C | MODERATE | 0.01(D) | 0.939(D) | NM_001109997.2 |
| *HEATR5A* | 14:31790773C>T | rs376940477 | p.R1301W | MODERATE | 0.01(D) | 0.968(D) | NM_015473.3 |
| *MYO9A* | 15:72338247G>A | N/A | p.D220N | MODERATE | 0(D) | 0.976(D) | NM_006901.3 |
| *TMC5* | 16:19498522T>C | N/A | p.L816P | MODERATE | 0(D) | 1.0(D) | NM_001105248.1 |
| *PSMB10* | 16:67968734C>T | N/A | p.R226W | MODERATE | 0(D) | 0.991(D) | NM_002801.3 |
| *TBC1D26* | 17:15641646G>T | rs201446121 | p.W111L | MODERATE | 0.01(D) | 0.992 (D) | NM_178571.4 |
| *KRTAP1-3* | 17:39191003G>C | N/A | p.C24S | MODERATE | 0.01(D) | 0.981(D) | NM_030966.1 |
| *CYP4F12* | 19:15806841A>C | N/A | p.Q404P | MODERATE | 0(D) | 0.932(D) | NM_023944.3 |
| *DHX34* | 19:47870293C>T | N/A | p.P550L | MODERATE | 0(D) | 0.758(P) | NM_014681.5 |
| *ZNF814* | 19:58384473A>G | N/A | p.H762R | MODERATE | 0(D) | 0.999(D) | NM_001144989.1 |
| *UBXN11* | 1:26608843G>T | rs6667693 | p.G504C | MODERATE | 0.01(D) | 0.992(D) | NM_183008.2 |
| *ADAM33* | 20:3654501C>T | rs182898006 | p.R266W | MODERATE | 0.02(D) | 0.994(D) | NM_025220.3 |
| *ZFP64* | 20:50701473C>T | N/A | p.R521C | MODERATE | 0(D) | 0.997 (D) | NM_199427.2 |
| *STK11IP* | 2:220471785G>A | rs17855575 | p.R393H | MODERATE | 0.03(D) | 0.982(D) | NM_052902.2 |
| *REG3A* | 2:79385823A>C | rs201139260 | p.H50P | MODERATE | 0.04(D) | 0.6(P) | NM_002580.2 |
| *RETSAT* | 2:85570849G>A | rs4832168 | p.G536R | MODERATE | 0(D) | 0.999(D) | NM_017750.3 |
| *CPB1* | 3:148563370C>G | N/A | p.P313R | MODERATE | 0(D) | 1.0(D) | NM_001871.2 |
| *SLC6A20* | 3:45814091C>T | N/A | p.A200V | MODERATE | 0(D) | 0.806(P) | NM_020208.3 |
| *FBXO43* | 8:101153493C>T | N/A | p.T330M | MODERATE | 0.01(D) | 0.974 (D) | NM_001029860.3 |
| *MTMR7* | 8:17166826G>A | N/A | p.E371K | MODERATE | 0(D) | 0.935(D) | NM_004686.4 |
| *WWP1* | 8:87393763T>A | N/A | p.F80Y | MODERATE | 0.05(D) | 0.999(D) | NM_007013.3 |
| *SCAI* | 9:127764259C>T | N/A | p.R400C | MODERATE | 0.02(D) | 0.828(P) | NM_173690.4 |
| *SMARCA1* | X:128633761C>T | N/A | p.P409S | MODERATE | 0(D) | 0.992(D) | NM_001282874.1 |
| *MTA3* | 2:42936041C>T | rs374558024 | p.R387C | MODERATE | 0(D) | 0.847(P) | NM_001282755.1 |
| *OCA2* | 15:28230211A>G | rs200764804 | p.R455G | MODERATE | 0(D) | 0.593(P) | NM_000275.2 |
| *SEMA4B* | 15:90771858C>T | N/A | p.R833C | MODERATE | 0(D) | 0.965(D) | NM_020210.3 |
| *PHKB* | 16:47545661A>G | rs144211929 | p.Y164C | MODERATE | 0(D) | 0.815(P) | NM_000293.2 |
| *BRCA1* | 17:41245262A>T | rs273898682 | p.R762S | MODERATE | 0(D) | 0.637(P) | NM_007300.3 |
| *MTHFR* | 1:11863038C>T | rs138189536 | p.R46W | MODERATE | 0.05(D) | 0.53(P) | NM_005957.4 |
| *FMO1* | 1:171249965G>A | rs16864310 | p.R227Q | MODERATE | 0.02(D) | 1.0(D) | NM_001282692.1 |
| *ITSN1* | 21:35183493C>T | rs200510636 | p.T845M | MODERATE | 0.01(D) | 0.927(D) | NM_003024.2 |
| *FAM98A* | 2:33810180G>T | rs199773047 | p.G407V | MODERATE | 0.01(D) | 0.909 (D) | NM_015475.3 |
| *PCDHGC3* | 5:140856752G>A | rs200686404 | p.V357M | MODERATE | 0.02(D) | 0.813(P) | NM_002588.3 |
| *C9* | 5:39308359T>C | N/A | p.C405R | MODERATE | 0(D) | 1.0(D) | NM_001737.3 |
| *CAPN11* | 6:44145138T>C | N/A | p.I466T | MODERATE | 0(D) | 0.971(D) | NM_007058.3 |
| *NUPL2* | 7:23235533A>G | rs199844379 | p.Y174C | MODERATE | 0.05(D) | 0.994(D) | NM_007342.2 |
| *THAP10* | 15:71184356G>A | rs184314334 | p.V86M | MODERATE | 0.01(D) | 0.889(P) | NM_020147.3 |
| *DHRS9* | 2:169940028G>A | rs146976196 | p.R228H | MODERATE | 0.03(D) | 0.993(D) | NM_001289763.1 |
| *CP* | 3:148895647G>A | rs187293972 | p.G1000S | MODERATE | 0(D) | 0.999(D) | NM_000096.3 |
| *PKN3* | 9:131469285C>T | rs138000368 | p.R212C | MODERATE | 0(D) | 0.999(D) | NM_013355.3 |
| *IFNW1* | 9:21141089G>T | rs201715468 | p.D161Y | MODERATE | 0(D) | 0.996(D) | NM_002177.1 |
| *ATXN3* | 14:92537353insC | N/A | p.G306Afs*12 | HIGH | N/A | N/A | NM_004993.5 |
| *IL32* | 16:3119297insG | rs71818662 | p.D172Gfs*12 | HIGH | N/A | N/A | NM_001012631.1 |
| *CDCP2* | 1:54605318insCC | rs3841798 | p.M409Pfs*2 | HIGH | N/A | N/A | NM_201546.3 |
| *SIGLEC1* | 20:3673567insGG  ATGAGGGTTTCT  ACAG | N/A | p.S1240_C1241ins  RDEGFYS | MODERATE | N/A | N/A | NM_023068.3 |
| *DYSF* | 2:71797784delGC  TGAGAAGATGTA  CTACACACACCG  ACGGCGGCGCTGG | N/A | p.A1048_W1060del | MODERATE | N/A | N/A | NM_001130987.1 |
| *IGSF10* | 3:151156346delCT | rs1176057739 | p.L2001Vfs*24 | HIGH | N/A | N/A | NM_178822.5 |
| *KCNN2* | 5:113698631insGCC | rs111266015 | p.A54_A55insAA | MODERATE | N/A | N/A | NM_021614.3 |
| *MAGEC1* | X:140996467insATAC | N/A | p.N1093Kfs*28 | HIGH | N/A | N/A | NM_005462.4 |
| *CD96* | 3:111261145insT | rs200703204 | p.V19Cfs*22 | HIGH | N/A | N/A | NM_198196.2 |
